# PRKAR1B as an oncogenic biomarker for diagnostic and prognostic stratification of tumor immunity, proliferation, and migration in head and neck squamous cell carcinoma

**DOI:** 10.3389/fimmu.2026.1770459

**Published:** 2026-02-20

**Authors:** Peng Zhao, Kang Li, WuFeng Xiu, Zhaokun Liu, Yanxiao Huang, Youfang Jiang, Peng Zhang, Lixiang Peng

**Affiliations:** 1 Department of Head & Neck Surgery, Jiangxi Cancer Hospital, The Second Affiliated Hospital of Nanchang Medical College, Jiangxi Clinical Research Center for Cancer, Nanchang, China; 2 Department of Otolaryngology, Shenzhen Longgang Otolaryngology Hospital & Shenzhen Institute of Otolaryngology, Shenzhen, China; 3 Quanzhou Orthopedic-Traumatological Hospital Affiliated to Fujian University of Traditional Chinese Medicine, Fujian, China; 4 Medical College of Nanchang University, Nanchang, China; 5 Department of Digestive Oncology, Jiangxi Cancer Hospital, The Second Affiliated Hospital of Nanchang Medical College, Jiangxi Clinical Research Center for Cancer, Nanchang, China

**Keywords:** hNSC, migration, PRKAR1B, proliferation, tumor immunity

## Abstract

Head and neck squamous cell carcinoma (HNSC) is one of the most prevalent malignancies worldwide. PRKAR1B, a regulatory component of protein kinase A (PKA), has been widely investigated for its potential involvement in tumorigenesis across different diseases. However, its specific role in HNSC remains elusive. In this study, significant differences in PRKAR1B expression were observed across various cancer types. PRKAR1B was highly expressed in HNSC and was strongly associated with poor prognosis in HNSC patients. Moreover, it was identified as an independent prognostic factor significantly associated with clinical parameters. Correlation analysis revealed that PRKAR1B expression was associated with genes such as C7orf50, EIF3B, TBRG4, DDX56, and BRAT1. Additionally, it was associated with TMB and was correlated with the infiltration of immune cells such as M1 macrophages, activated mast cells, and eosinophils. Notably, PRKAR1B was identified as a predictive marker for the efficacy of CTLA-4 inhibitors, with high PRKAR1B expression potentially conferring superior therapeutic responses. Drug sensitivity analysis further suggested that Lapatinib and Erlotinib may be beneficial in HNSC patients with high PRKAR1B expression. Meanwhile, *in vitro* experiments showed that PRKAR1B knockdown inhibited HNSC cell proliferation and migration. Lastly, PRKAR1B protein expression was upregulated in clinical HNSC samples. Overall, this study thoroughly examined PRKAR1B expression and its prognostic significance in HNSC, investigated related molecular pathways and immune cell interactions, and validated its role via *in vitro* experiments.

## Introduction

As is well documented, head and neck squamous cell carcinoma (HNSC) is one of the most prevalent cancers globally (1, 2). Several risk factors for HNSC development have been identified, such as carcinogen exposure (e.g., smoking, alcohol consumption, betel nut chewing, air pollution), candidiasis, Epstein-Barr virus, and human papillomavirus (3, 4). At present, HNSC is generally managed with surgical resection, followed by adjuvant radiotherapy or chemoradiotherapy based on disease stage, with chemoradiotherapy remaining a primary treatment strategy for cancers arising in the pharynx or larynx (5).

Over half of HNSC patients are diagnosed at a locally advanced stage at their initial presentation and are typically treated with surgery, radiotherapy, and systemic therapy (6). HNSC unsuitable for curative surgery or radiotherapy is generally managed with palliative systemic regimens, including platinum-based chemotherapy, cetuximab, and immune checkpoint inhibitors (ICIs) targeting PD-1. Despite these approaches, quality of life is severely compromised, and treatment efficacy remains suboptimal, highlighting the urgent need for novel therapies with reduced toxicity and improved effectiveness (7, 8).

Cyclic adenosine monophosphate (cAMP)-dependent protein kinase A (PKA) and protein phosphatase 1 (PP1) are crucial multifunctional proteins involved in signaling pathways that modulate physiological and pathological processes associated with cancer development and progression (9). The PRKAR1B gene encodes the β regulatory subunit of PKA implicated in the cAMP signaling pathway. PKA is a holoenzyme consisting of two regulatory and two catalytic subunits that dissociate upon cAMP binding (10). This holoenzyme participates in various cellular processes such as ion transport, metabolism, and transcription (11). Located on chromosome 7p22, PRKAR1B exhibits multiple transcript variants encoding the same protein and is implicated in cancer initiation and progression. Thus, this study aimed to investigate the role of PRKAR1B in HNSC progression.

## Materials and methods

### Gene expression analysis of PRKAR1B in pan-cancer

PRKAR1B RNA and protein expression profiles in healthy human tissues were collected from the HPA database (https://www.proteinatlas.org/). Pan-Cancer RNA-Seq was obtained from the TCGA (https://portal.gdc.cancer.gov/) and GTEx (https://commonfund.nih.gov/GTEx) databases. PRKAR1B mRNA expression in tumor and adjacent normal samples from the TCGA database was examined using the Timer2.0 platform (http://timer.cistrome.org/). Matched normal tissue expression data from the GTEx database were also analyzed to offer a comprehensive overview. Radar plots were generated, and survival analyses, including proportional hazards assumption testing and Cox regression, were conducted using the survival package in R. Immune infiltration scores were calculated using the ssGSEA algorithm in the GSVA R package.

### Survival analysis

Cases lacking complete clinical data were excluded, and gene expression values were categorized into high and low groups using R. Kaplan-Meier survival analysis was conducted, and proportional hazards assumptions were evaluated using the survival package. Survival regression models were applied, and the ‘survminer’ and ‘ggplot2’ packages were employed to generate Kaplan-Meier curves. The predictive performance of PRKAR1B in HNSC patients was evaluated using ROC curve analysis implemented in the “pROC” R package.

### Identification of PRKAR1B-related genes

To identify genes co-expressed with PRKAR1B, transcriptomic data from the TCGA-HNSC cohort were analyzed using the “limma” package in R. Pearson correlation coefficients were calculated, with significance defined as *p* < 0.001. Co-expressed genes were visualized using heatmaps.

### Functional Enrichment Analysis

In the TCGA cohort, HNSC patients were categorized into high- and low-PRKAR1B expression groups using the median PRKAR1B expression level as the threshold. Differentially expressed genes (DEGs) were determined using the ‘limma’ package in R, applying thresholds of logFC > 1 and p < 0.05 (12). DEGs were visualized using heatmaps, and functional enrichment analyses, such as Gene Ontology (GO) and Kyoto Encyclopedia of Genes and Genomes (KEGG), were conducted using the ‘clusterProfiler’ R package (13).

### Tumor-infiltrating immune cells in HNSC

The relative proportions of TIICs in HNSC samples were assessed using the CIBERSORT algorithm. Immune infiltration scores for 22 TIICs were derived from gene expression matrices and compared against the CIBERSORT reference matrix using 1,000 permutations. Wilcoxon tests were utilized to assess differences in immune infiltration between the high- and low-PRKAR1B expression groups. The relationship between PRKAR1B expression and immune checkpoint-related gene levels was examined using Pearson correlation analysis.

### Immune assay

The relationship between PRKAR1B and immune phenotypes was evaluated to assess potential sensitivity to immune therapy. Pearson correlation analysis was carried out to evaluate relationships between PRKAR1B and 47 immune checkpoint-related genes. A threshold of p < 0.001 was set for statistical significance. Tumor mutational burden (TMB) for each sample was determined using the TMB function in R, which combined TMB data with gene expression data. Pearson correlation was employed to evaluate the association between TMB and gene expression across tumors. The pRRophetic R package, utilizing drug sensitivity data from the Cancer Genome Project, was used to predict chemotherapeutic drug sensitivity, expressed as IC50 values. The Wilcoxon test was applied to compare IC50 values between the high- and low-PRKAR1B expression groups. Immunophenoscores (IPS), sourced from the TCIA website (https://tcia.at/home), have demonstrated predictive capability for patient responses to ICI therapy (14).

### Single-cell analysis

Single-cell data from the Tumor Immune Single-Cell Hub (TISCH2) database (GSE103322) were analyzed to investigate PRKAR1B distribution at the single-cell level. UMAP was employed for cell clustering, annotation, and visualization (15).

### Cell culture

HNSC cell lines SCC9 and SCC25 were procured from BNCC (BeNa Cell Culture Collection). SCC9 cells were maintained in DMEM supplemented with 10% fetal bovine serum, 100 μg/mL penicillin, and streptomycin, whereas SCC25 cells were maintained in DMEM-H/F-12K supplemented with 10% FBS and 400 ng/mL hydrocortisone. Cells were incubated at 37 °C in a humidified atmosphere with 5% CO_2_.

### Transfection

Cells were plated in 6-well plates, then PRKAR1B siRNA and GP-transfect-Mate were supplied by GenePharma Co., Ltd., and transfection followed the manufacturer’s guidelines. The siRNA sequences used were as follows: NC siRNA: 5′-GCTTCGCGCCGTAGTCTTATCA-3′, PRKAR1B siRNA: 5′-CGUCCAGUUUGAAGAUGGATT-3′.

### RT-qPCR

RNA was isolated from treated cells using the RNeasy Mini Kit (Qiagen, 74104). cDNA was synthesized using the RT Master Mix Kit (MedChemExpress, HY-K0511). qPCR was conducted on a 7500 fast real-time PCR system utilizing SYBR Green qPCR Master Mix (MedChemExpress, HYK0522). Each reaction was conducted in a final volume of 20 μL. The primers utilized were as follows: β-actin forward primer 5′-CACCATTGGCAATGAGCGGTTC-3′ and reverse primer 5′-AGGTCTTTGCGGATGTCACCGT-3′; PRKAR1B forward primer 5′-CAGGTCCTCAAAGACTGTATCGT-3′ and reverse primer 5′-ATGGGAGTCCGACTGTGAGT-3′; E-cadherin forward primer 5′-GCCTCCTGAAAAGAGAGTGGAAG-3′ and reverse primer 5′-TGGCAGTGTCTCTCCAAATCCG-3′; N-cadherin forward primer 5′-CCTCCAGAGTTTACTGCCATGAC-3′ and reverse primer 5′-GTAGGATCTCCGCCACTGATTC-3′.

### Western blotting

Equal amounts of protein were separated using 10% SDS-PAGE and transferred onto PVDF membranes. Next, the membranes were blocked with 5% BSA in TBST for 1 hour at room temperature, followed by overnight incubation at 4 °C with anti-PRKAR1B (1:1000, Proteintech) and anti-β-actin (1:3000, Santa Cruz Biotechnology). Afterward, they were incubated with HRP-conjugated secondary antibodies (Cell Signaling) at room temperature for 2 hours. Protein bands were analyzed using ImageJ software.

### Immunofluorescence

Cells were fixed with 4% paraformaldehyde for 20 minutes and subsequently permeabilized using 0.1% Triton X-100 for 30 minutes. They were then incubated overnight at 4 °C with the primary antibody against PRKAR1B (1:200, Proteintech) in confocal Petri dishes. Thereafter, cells were incubated at room temperature with Plus 555-Goat Anti-Rabbit Recombinant Secondary Antibody (H+L) at a 1:500 dilution (Proteintech) for 2 hours. Finally, the cells were then stained with DAPI for 30 minutes.

### CCK8 assay

CCK8 assays were conducted following the instructions provided by MedChemExpress. Briefly, cells were cultured for 0, 24, 48, and 72 hours. Following this, 10 μL of CCK-8 reagent was added to each well for 2 hours at 37 °C. Cell viability was evaluated by measuring absorbance at OD 450 nm.

### EdU labeling assay

Cell proliferation was assessed following siRNA transfection using the BeyoClick™ EdU Cell Proliferation Kit with AF488.

### Transwell assay

Transfected SCC9 and SCC25 cells (2 × 10^4^) were seeded into the upper chamber of Transwell inserts in 200 μL of serum-free DMEM, while the lower chamber contained 600 μL of DMEM supplemented with 10% FBS. After incubation, cells were fixed with 4% paraformaldehyde and stained with crystal violet. Non-migratory cells in the upper lumen were removed.

### Wound healing assay

Cells were seeded in six-well plates and cultured until approximately 90% cell confluence. Afterward, a linear scratch was created in each well using a sterile 20 μL plastic pipette tip, following which cells were incubated in serum-free medium for 24 hours. Images of the wound area were captured at 0 and 24 hours and visualized under an inverted microscope.

### Immunohistochemistry staining

Surgical paraffin-embedded tissue specimens were obtained from Jiangxi Cancer Hospital. Inclusion criteria: Pathologically confirmed primary head and neck squamous cell carcinoma; no prior surgery, radiotherapy or chemotherapy before admission; no history of head and neck tumors; complete data. Exclusion criteria: Radiotherapy, chemotherapy or other clinical interventions prior to surgery; presence of distant metastasis at initial diagnosis; severe organ dysfunction; autoimmune diseases, hematological diseases, or history of other malignant tumors; incomplete data. Immunohistochemistry staining was performed as previously described. The German semi-quantitative scoring system was used in this study.

### Statistical analysis

Statistical analyses were conducted using R software version 4.2.3 and GraphPad Prism version 7.00. Data were analyzed using standard statistical tests. Two-tailed P values were reported, with p < 0.05 considered statistically significant.

## Results

### Differential expression of PRKAR1B in pan-cancer and normal tissues

**Figure 1:**
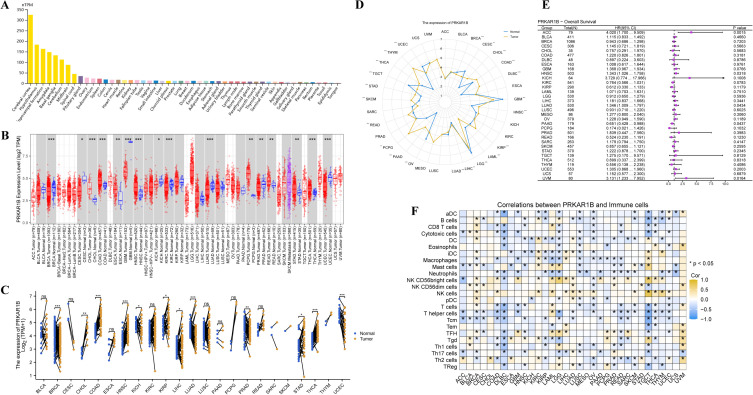
Expression of PRKAR1B under physiological conditions and across cancers. **(A)** PRKAR1B expression across different tissues under physiological conditions. **(B)** Analysis of PRKAR1B expression levels between tumor and normal tissues across multiple cancer types using the TIMER database. **(C)** Further analysis of PRKAR1B expression in various tumors and paired adjacent normal tissues using the TCGA database. **(D)** Radar plot displaying PRKAR1B expression in the TCGA and GTEx joint cohort. **(E)** Forest plot showing the prognostic significance of PRKAR1B across different tumors within the TCGA cohort. **(F)** Heatmap illustrating the correlation between PRKAR1B expression and the infiltration levels of various immune cell subsets across tumors. *P < 0.05; **P < 0.01; ***P < 0.001.

The expression of PRKAR1B in various tissues under physiological conditions was analyzed using the HPA database. As anticipated, PRKAR1B expression was the highest in the cerebral cortex (**Figure 1A**). In addition, the TIMER database was employed to assess PRKAR1B expression across 33 tumor types and corresponding normal tissues from the TCGA database. The results revealed that PRKAR1B mRNA expression levels were reduced in Breast invasive carcinoma (BRCA), Cervical squamous cell carcinoma and endocervical adenocarcinoma (CESC), Kidney chromophobe (KICH), Kidney renal clear cell carcinoma (KIRC), Lung adenocarcinoma (LUAD), Prostate adenocarcinoma (PRAD), and Uterine corpus endometrial carcinoma (UCEC) compared to normal tissues. On the other hand, elevated PRKAR1B mRNA expression was detected in several cancers, including Head and Neck Squamous Cell Carcinoma (HNSC), Cholangiocarcinoma (CHOL), Colon Adenocarcinoma (COAD), Esophageal Carcinoma (ESCA), Glioblastoma Multiforme (GBM), Pheochromocytoma and Paraganglioma (PCPG), Thyroid Carcinoma (THCA), Stomach Adenocarcinoma (STAD), and Rectum Adenocarcinoma (READ) (**Figure 1B**). These findings indicated that mRNA levels of PRKAR1B were higher in tumor tissues compared to adjacent normal tissues across various cancers, including COAD, HNSC, KICH, KIRP, LIHC, LUAD, PRAD, STAD, THCA, and CHOL (**Figure 1C**). To address the absence of normal tissue data for some tumors in the TIMER database, the TCGA and GTEx datasets were integrated, and radar charts were plotted to explore PRKAR1B expression in 33 tumor types. Interestingly, the analysis showed that PRKAR1B expression was up-regulated in 15 tumor types compared to their corresponding normal tissues (**Figure 1D**). To investigate the prognostic relevance of PRKAR1B expression, Cox regression survival analysis was performed using the TCGA cohort. Importantly, significant associations were observed in HNSC, ACC, LUAD, PAAD, and UVM. In HNSC, the hazard ratio (HR) exceeded 1 (95% CI: 1.026-1.758), positioning PRKAR1B as a potential risk factor (**Figure 1E**). Besides, the ssGSEA was applied to assess the infiltration of 24 immune cell types within the TCGA cohort, revealing significant correlations between PRKAR1B expression and T cell subsets, NK cells, and B cells, highlighting its potential role in the tumor microenvironment (**Figure 1F**).

### Prognostic value of PRKAR1B in HNSC

**Figure 2:**
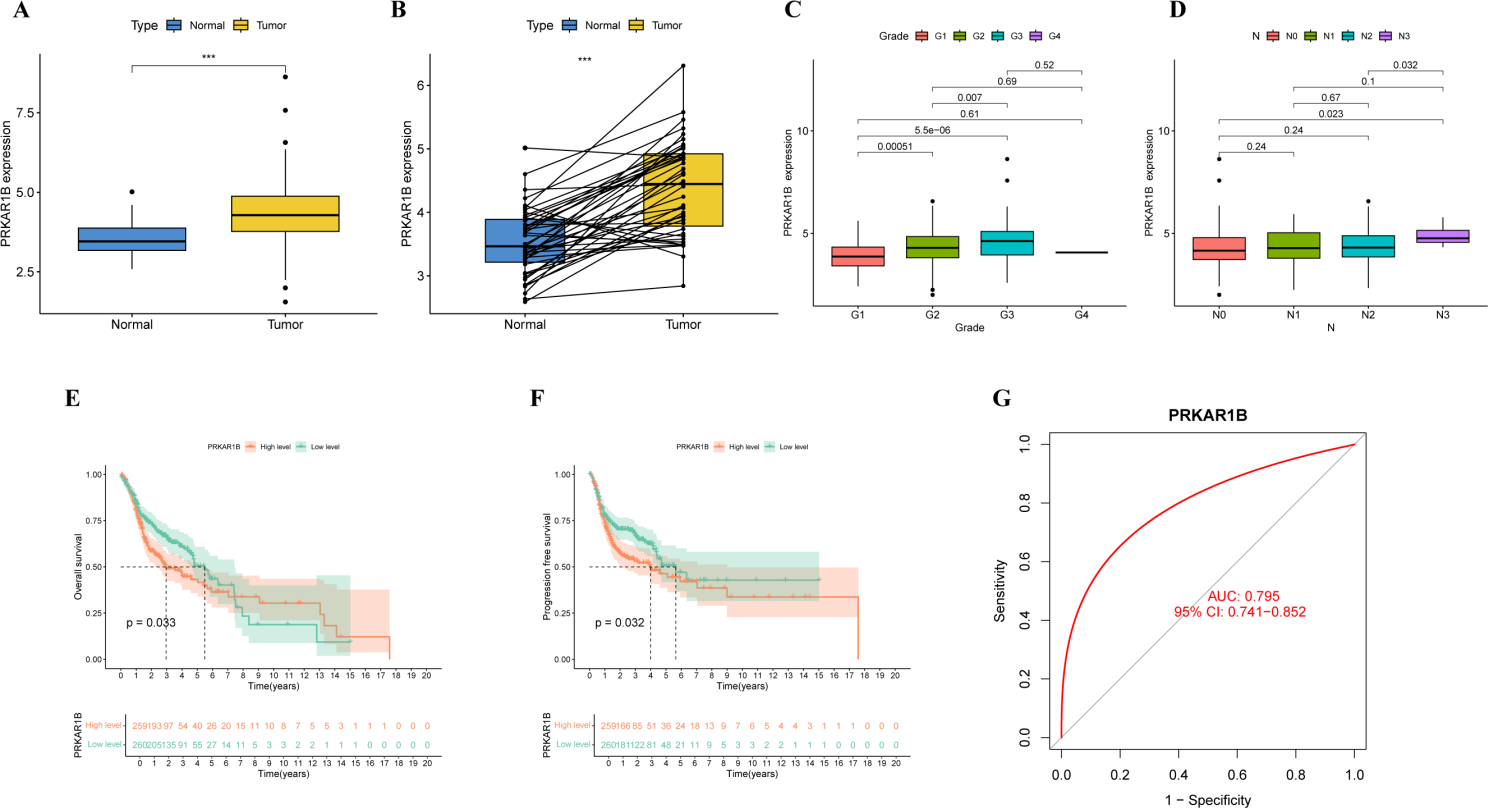
Prognostic value of PRKAR1B in HNSC patients. **(A, B)** PRKAR1B expression in HNSC tumor tissues and paired and non-paired normal tissues from the TCGA database. **(C)** Correlation between PRKAR1B expression and tumor grade and N stage in HNSC patients. **(D)** Kaplan-Meier survival curves delineating differences in OS and PFS between high- and low-PRKAR1B expression groups in HNSC patients. **(E, F)** OS and PFS survival curves. **(G)** ROC curve analysis showing the diagnostic value of PRKAR1B in HNSC. *** P <0.001.

In the present study, PRKAR1B expression was analyzed in 566 samples derived from the TCGA-HNSC cohort. The analysis revealed significantly elevated PRKAR1B expression levels in HNSC tumor tissues compared to normal and paired adjacent tissues (**Figures 2A, B**). However, there was no significantly PRKAR1B expressions between HPV-positive and HPV-negative tumor tissues (**Supplementary Figure 1**). To assess the clinical relevance of PRKAR1B expression, its correlation with various clinical factors was examined in HNSC patients, unveiling that PRKAR1B expression was correlated with tumor grade and N-stage (**Figures 2C, D**). Survival analysis revealed that higher PRKAR1B expression levels were correlated with poorer overall survival (OS) and progression-free survival (PFS) in patients with HNSC (P < 0.05). Furthermore, Kaplan-Meier survival curves illustrated that elevated PRKAR1B expression levels were correlated with poorer prognosis, implying its significant role in tumor progression (**Figures 2E, F**). Additionally, ROC analysis, performed to explore the diagnostic value of PRKAR1B level in HNSC patients, indicated an AUC of 0.795, thereby supporting its significant diagnostic potential in HNSC (**Figure 2G**).

### Screening of co-expressed genes with PRKAR1B

**Figure 3:**
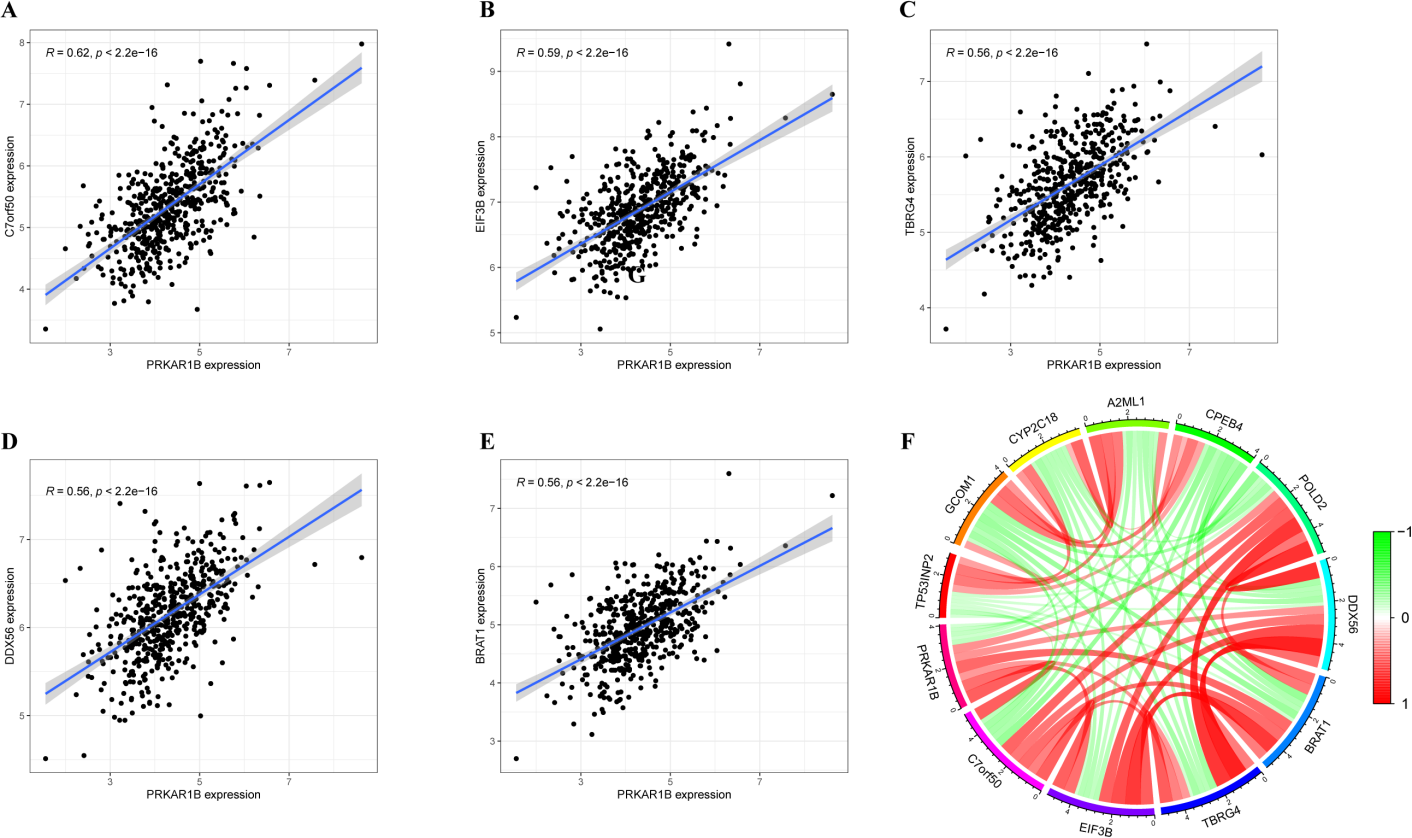
Genes co-expressed with PRKAR1B. **(A-E)** The top five genes co-expressed with PRKAR1B \in the TCGA-HNSC. **(F)** Chord diagram depicting the top ten genes co-expressed with PRKAR1B.

Correlation analysis of the TCGA-HNSC dataset was performed to identify genes co-expressed with PRKAR1B. The top five co-expressed genes, namely C7orf50, EIF3B, TBRG4, DDX56, and BRAT1, were identified (**Figures 3A**). Of note, these genes have been extensively studied in various diseases. A chord diagram was established to visualize the relationship between genes whose expression correlated positively or negatively with PRKAR1B expression, displaying the top twelve genes with significant positive or negative correlations with PRKAR1B expression (**Figure 3F**). These genes may directly or indirectly affect PRKAR1B expression or may be regulated by PRKAR1B.

### Biological functions of PRKAR1B in HNSC

**Figure 4:**
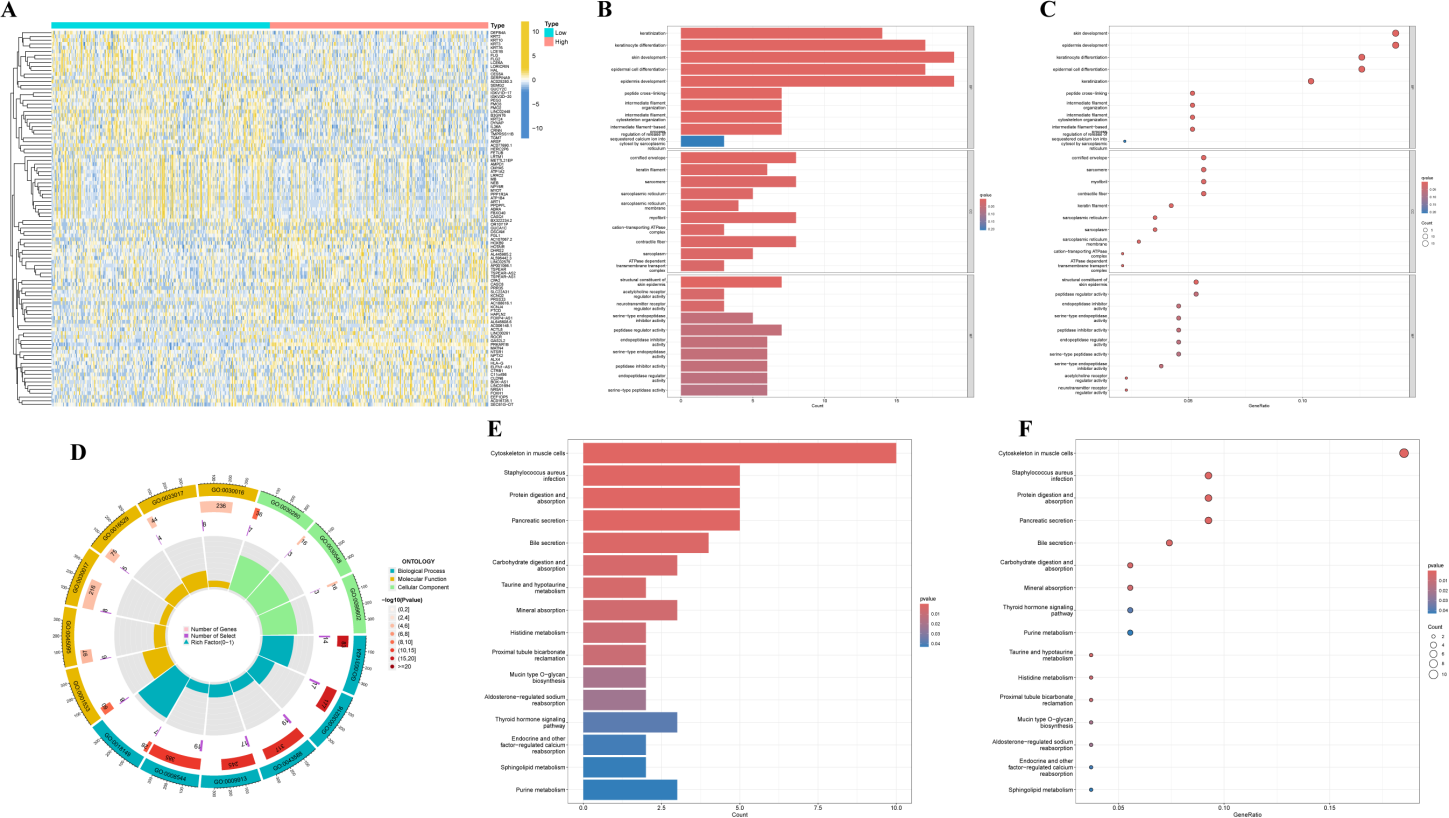
Biological functions of PRKAR1B in HNSC. **(A)** Heatmap depicting the top fifty differentially expressed genes between high- and low-PRKAR1B expression groups. **(B-D)** Bar plot, bubble plot, and circle plot depicting the results of GO enrichment analysis for the differentially expressed genes. **(E, F)** Bar plot and bubble plot showing KEGG pathway enrichment for the differentially expressed genes.

Differential expression analysis was performed on HNSC tumor samples stratified by PRKAR1B expression to explore its molecular role in tumorigenesis and progression. The top 50 DEGs were depicted in a heatmap (**Figure 4A**). GO and KEGG pathway enrichment analyses were conducted on these DEGs. GO functional analysis revealed that PRKAR1B is involved in skin development, epidermal development, keratinocyte differentiation, and epidermal cell differentiation (**Figures 4B**). At the same time, KEGG pathway analysis uncovered enrichment in several tumor-related KEGG pathways, including Cytoskeleton in muscle cells, Protein digestion and absorption, Pancreatic secretion, and Staphylococcus aureus infection (**Figures 4E, F**).

### Immune landscape and PRKAR1B in HNSC

**Figure 5:**
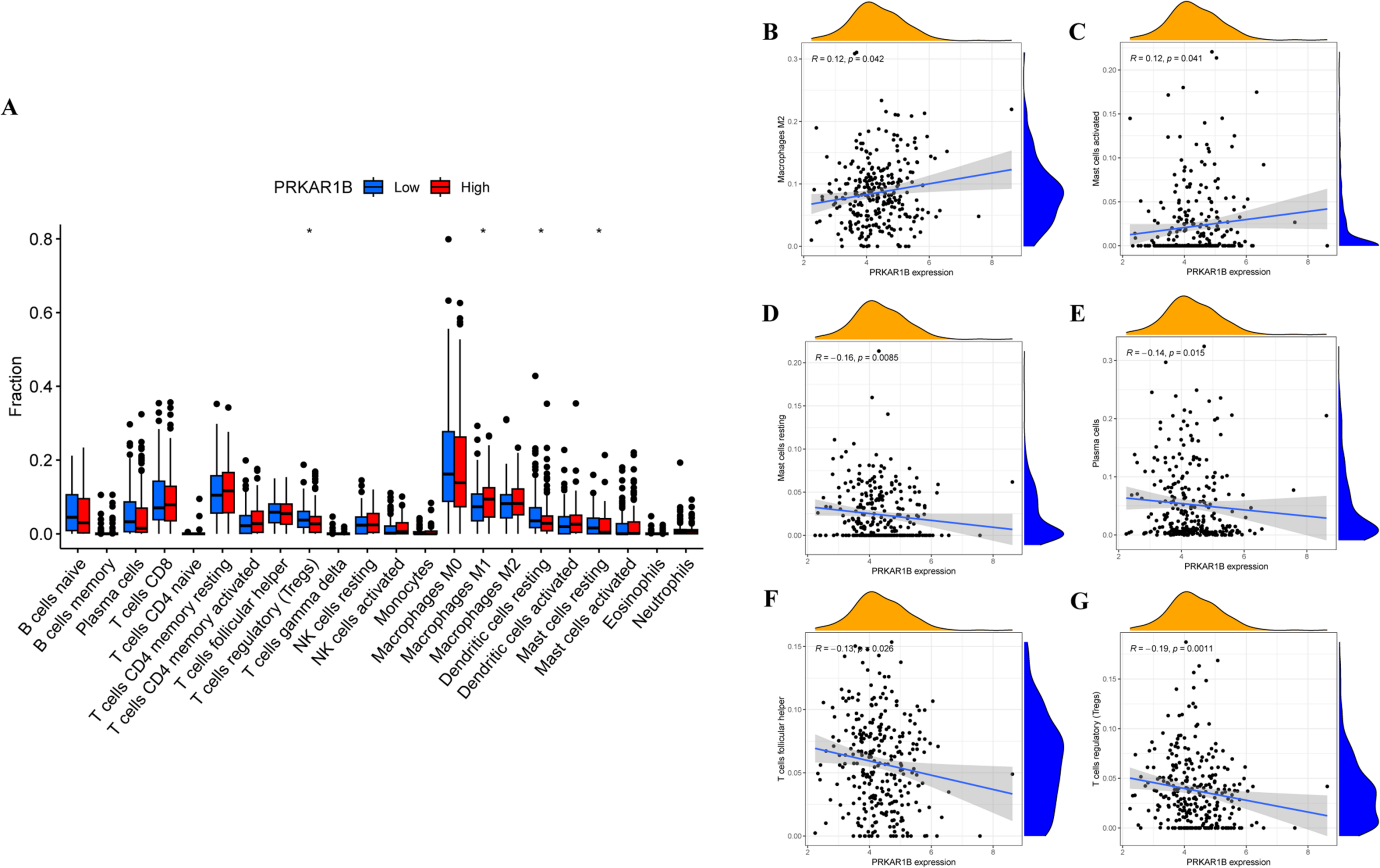
Correlation between PRKAR1B and immune infiltration in HNSC. **(A)** Immune cell infiltration levels between high- and low-PRKAR1B expression groups. **(B-G)** Correlation between PRKAR1B expression and immune cell infiltration across immune cell types. *P < 0.05.

**Figure 6:**
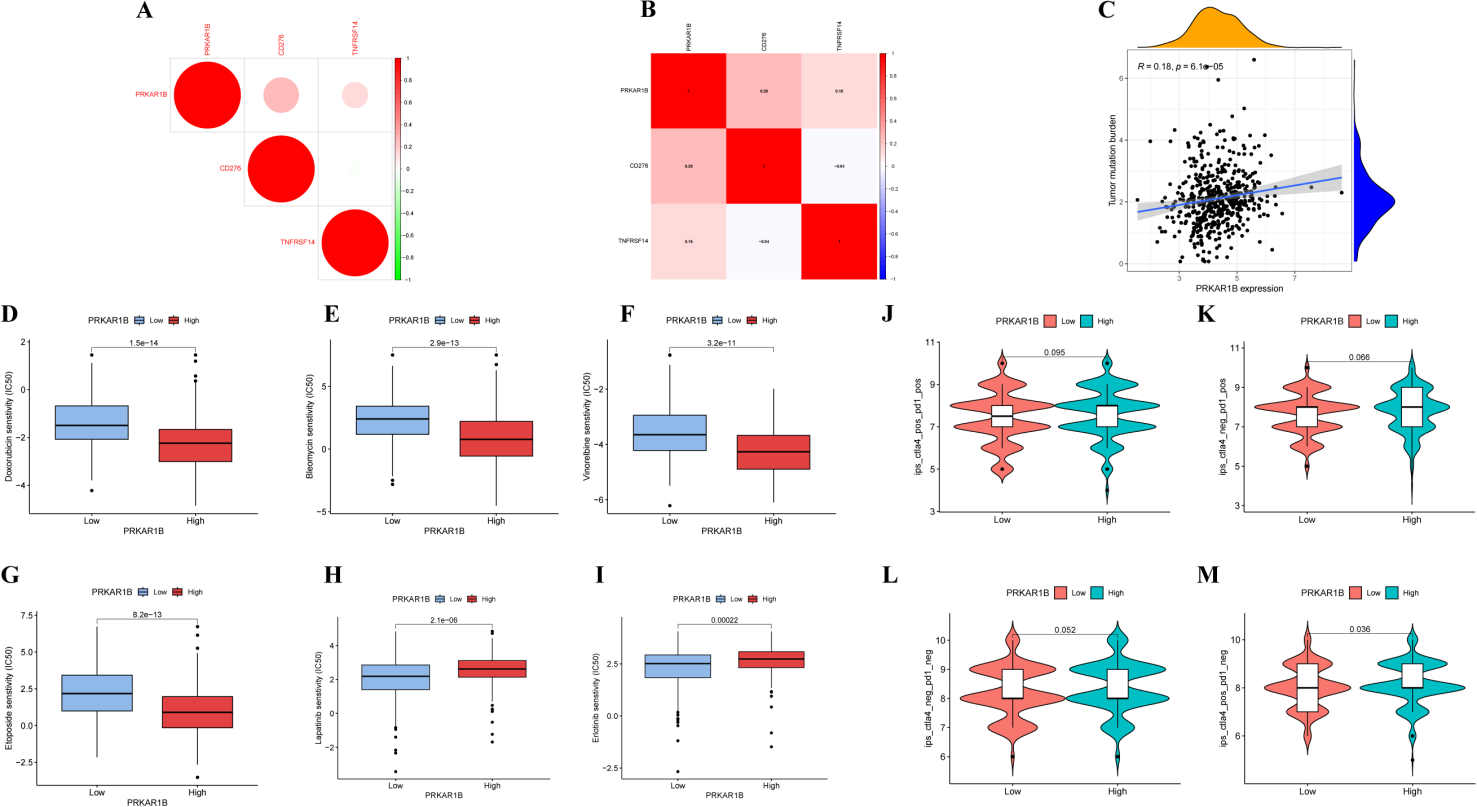
Correlation between PRKAR1B and immune therapy in HNSC patients. **(A, B)** Correlation between PRKAR1B expression and immune checkpoint genes. **(C)** Correlation between PRKAR1B expression and TMB. **(D-I)** Chemotherapeutic drugs showing differential sensitivity in high- and low-PRKAR1B expression groups based on IC50 analysis. **(J-M)** PRKAR1B expression as a predictor of therapeutic response to immune checkpoint inhibitors.

The CIBERSORT algorithm was adopted to evaluate correlations between PRKAR1B expression and the infiltration of different immune cell subsets. The results revealed significant differences in immune infiltration between the high- and low-PRKAR1B expression groups, especially for resting mast cells, resting dendritic cells, Tregs, and M1 macrophages (**Figure 5A**). Specifically, PRKAR1B expression was positively correlated with the infiltration of M2 macrophages and activated mast cells, as well as negatively correlated with the infiltration of resting mast cells, plasma cells, follicular helper T cells, and Tregs (**Figure 5B**). To explore the impact of PRKAR1B on immunotherapy, its relationship with immune checkpoint genes was examined, revealing correlations between PRKAR1B expression levels and the immune checkpoint genes CD276 and TNFRSF14 (**Figures 6A, B**). TMB, defined as the total count of base mutations per million tumor cells, is recognized for inducing tumor-specific, highly immunogenic antibodies and is emerging as a promising biomarker for immunotherapy response in cancer patients (16). Noteworthily, PRKAR1B expression showed a significant correlation with TMB in HNSC (**Figure 6C**). Additionally, IC50 values for various chemotherapeutic drugs were evaluated based on PRKAR1B expression levels using data derived from the Cancer Genome Project database. Lapatinib and Erlotinib exhibited greater efficacy in the high-PRKAR1B expression group, whereas Bleomycin, Doxorubicin, Vinorelbine, and Etoposide were more effective in the low-PRKAR1B expression group. These findings collectively suggest that PRKAR1B expression may predict chemotherapy responses in HNSC, reinforcing its potential as a biomarker for personalized treatment strategies (**Figure 6D**). Immune checkpoint genes (ICGs) are determinants of immunotherapy efficacy, and analyzing the clinical data and expression of several ICGs can assist in the identification of potential therapeutic targets for personalized treatment. To explore the specific impact of PRKAR1B on immunotherapy, the effect of ICIs on PRKAR1B was explored. Immunophenoscores were utilized to predict patient responses to different immune checkpoint inhibitor combinations. High PRKAR1B expression was associated with a higher IPS for anti-CTLA4 therapy, signaling that high PRKAR1B expression may correlate with improved immune response to ICI therapy (**Figure 6J**).

### Single-cell analysis of PRKAR1B

**Figure 7:**
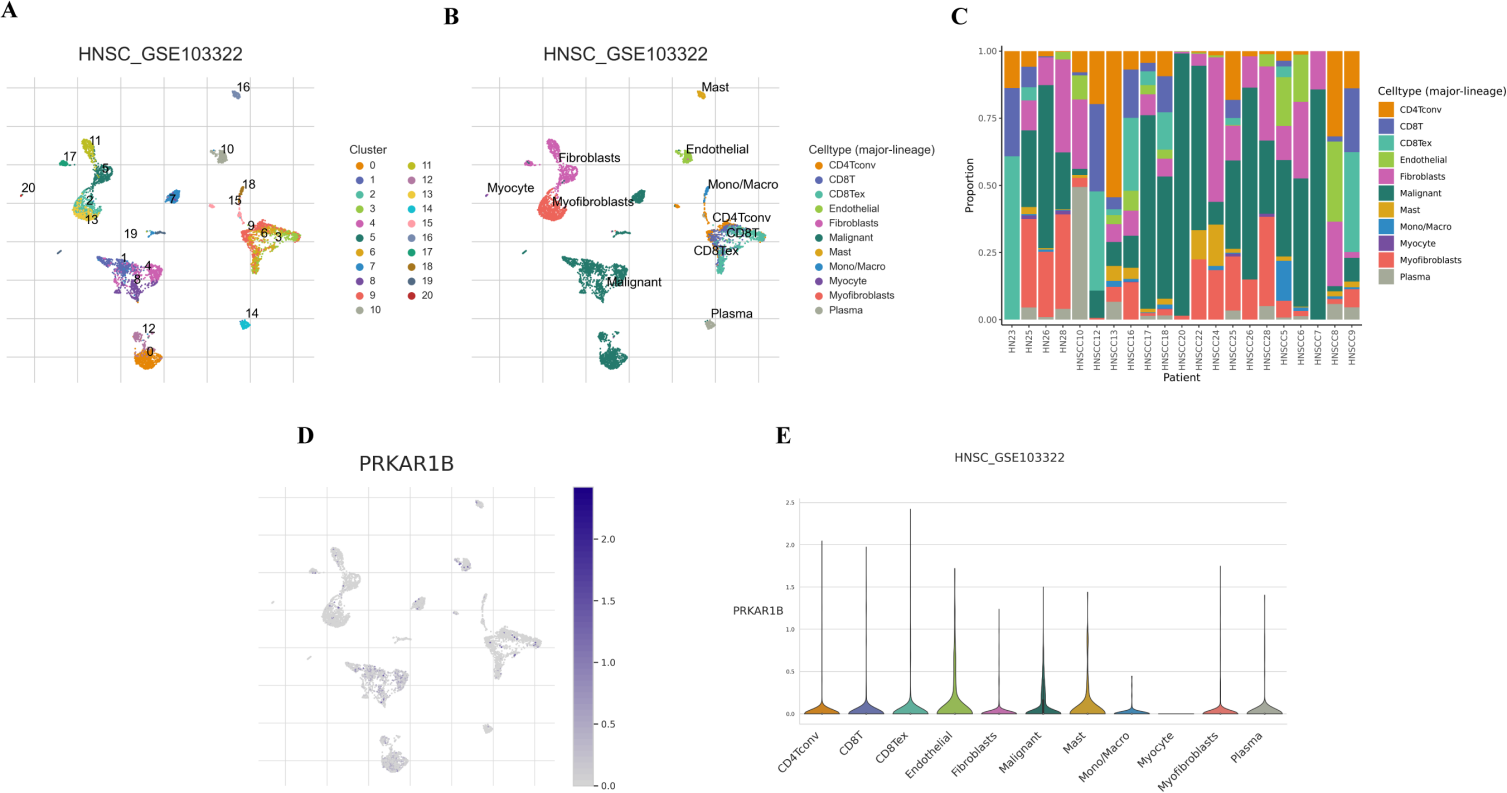
PRKAR1B expression in different cell populations in HNSC. **(A)** UMAP clustering of cells from the HNSC_GSE103322 dataset. **(B)** UMAP clustering of 11 cell types in the GSE103322 dataset. **(C-E)** Expression patterns of PRKAR1B across cell types.

Furthermore, the distribution of PRKAR1B was analyzed using single-cell transcriptome data from GSE103322. A total of 20 cell clusters were identified and categorized into 11 distinct cell types based on marker gene expression: CD4Tconv, CD8+ T cells, CD8Tex, Endothelial cells, Fibroblasts, Malignant cells, Mast cells, Mono/Macro cells, Myocytes, Myofibroblasts, and Plasma cells (**Figures 7A, B**). PRKAR1B was highly expressed in CD8Tex, Endothelial, and Mast cells, whereas lower expression levels were detected in Myocytes (**Figures 7C, D**).

### PRKAR1B knockdown inhibits HNSC cell proliferation and migration

**Figure 8:**
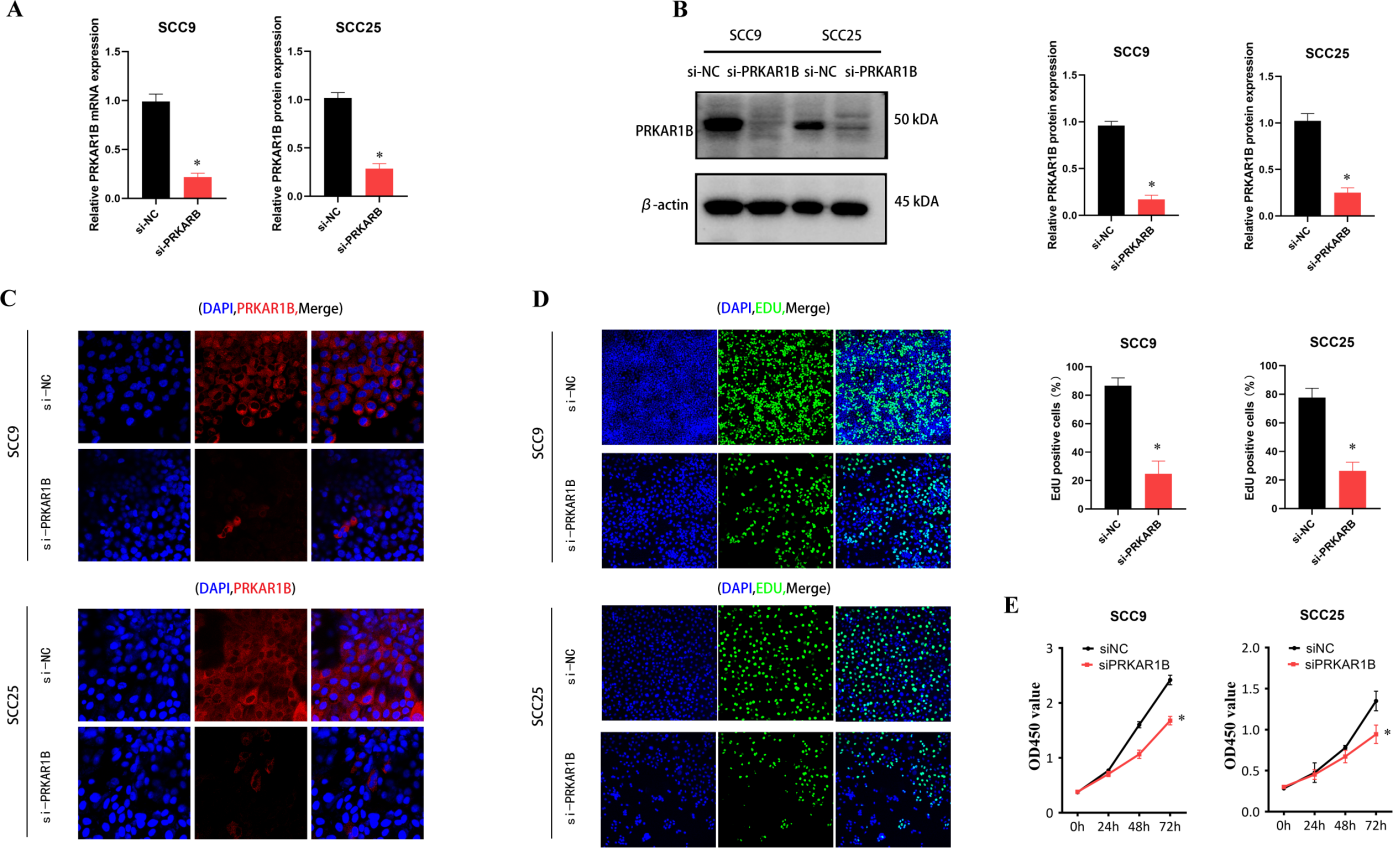
PRKAR1B knockdown inhibits HNSC cell proliferation. **(A-C)** Western blot, RT-qPCR, and immunofluorescence demonstrating the knockdown efficiency of PRKAR1B in SCC9 and SCC25 cell lines. **(C, D)** CCK8 and EdU labeling assays showing that PRKAR1B knockdown inhibited the proliferation of SCC9 and SCC25 cells. **(E)** CCK-8 assay showing the proliferative capacity of SCC9 and SCC25 with PRKAR1B knockdown. *P < 0.05.

**Figure 9:**
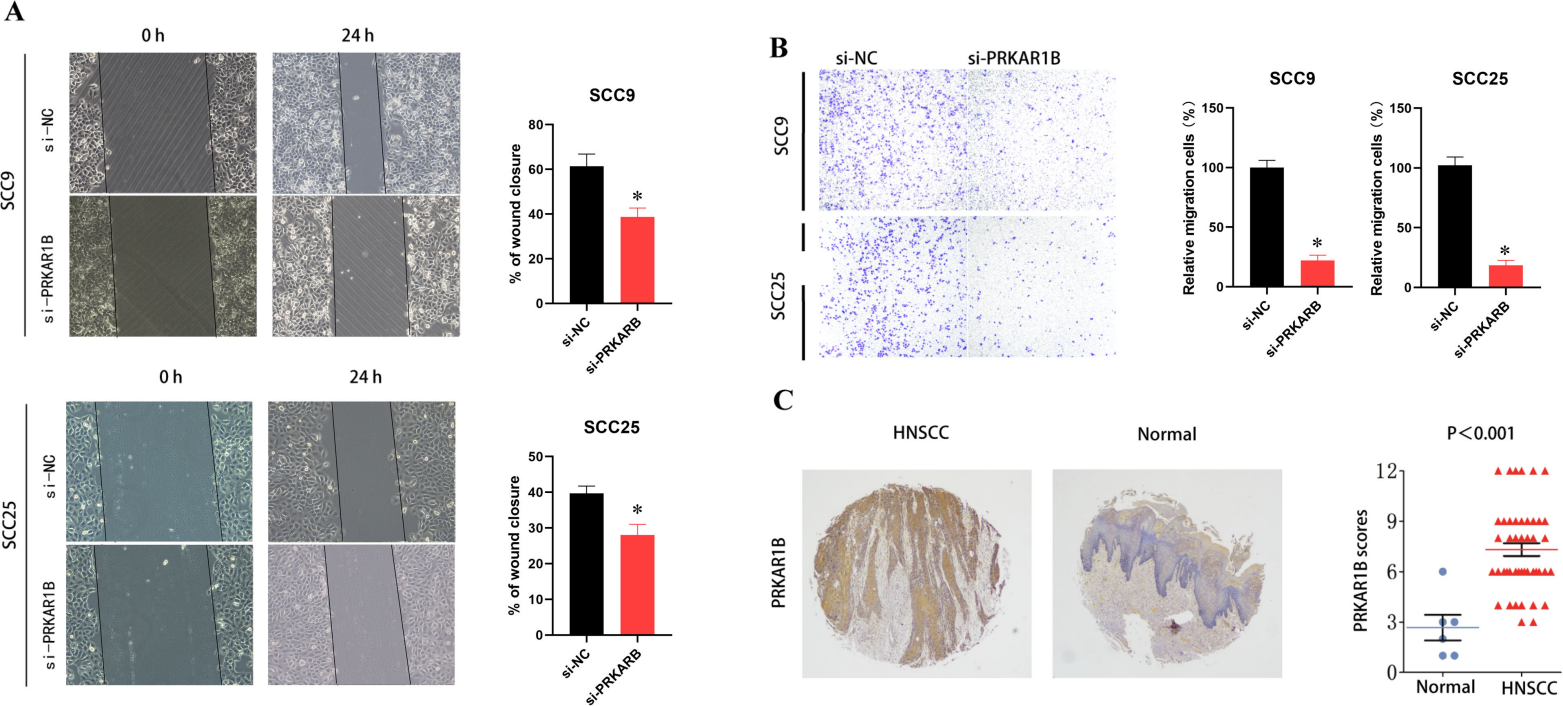
PRKAR1B knockdown inhibits the migratory abilities of HNSC cells. **(A, B)** Wound-healing and Transwell assays demonstrating the inhibitory effect of PRKAR1B knockdown on the migratory capabilities of SCC9 and SCC25 cells. **(C)** IHC demonstrating high PRKAR1B expression in HNSC tissues. *P < 0.05.

To validate the role of PRKAR1B in HNSC, siRNA was used to knockdown its expression in SCC9 and SCC25 cells. Transfection efficiency was validated by RT-qPCR (**Figure 8A**) and Western blotting (**Figure 8B**) and further validated by immunofluorescence. Notably, upon transfection of PRKAR1B siRNA, significantly reduced PRKAR1B levels were observed in both SCC9 and SCC25 cells (**Figure 8C**). CCK8 and EdU labeling assays demonstrated that PRKAR1B knockdown inhibited the proliferative abilities of SCC9 and SCC25 cells (**Figures 8C, D**). In addition, Transwell and wound healing assays showed that PRKAR1B silencing significantly suppressed the migratory abilities of SCC9 and SCC25 cells compared with controls. Taken together, these results confirmed that PRKAR1B knockdown may suppress the metastatic capabilities of SCC9 and SCC25 cells (**Figures 9A, B**). Moreover, PRKAR1B knockdown upregulated E-cadherin expression and downregulated N-cadherin expression in SCC9 and SCC25 cells (**Supplementary Figure 2**). Finally, immunohistochemical staining was performed to examine PRKAR1B protein expression levels in both HNSC and normal tissue samples, revealing that PRKAR1B protein was highly expressed in HNSC tissues (**Figure 9C**).

## Discussion

To date, treatment options for HNSC are largely restricted to regional surgery, conventional chemotherapy, and radiotherapy. Despite improvements in clinical expertise and advancements in medical technology, treatment outcomes remain unsatisfactory, and the prognosis for HNSC remains suboptimal. The modest improvement in survival rates over decades is primarily attributed to late-stage diagnoses, the limited efficacy of targeted therapies, and the absence of reliable biomarkers. Prevention, early detection, and biomarker-driven therapeutic adjustments are crucial for timely intervention and optimizing clinical outcomes (17). Recent studies have concluded that PRKAR1B significantly contributes to cancer progression. In ovarian cancer, PRKAR1B is implicated in cisplatin resistance, cross-resistance to paclitaxel, and cisplatin-induced metastasis (18). Additionally, PRKAR1B may contribute to the development of adrenal cortical disease through cAMP signaling disruption, thereby promoting kidney tumorigenesis (19). Overall, this study validates the role of PRKAR1B in the progression of HNSC and positions it as a diagnostic biomarker for HNSC.

Bioinformatics analysis revealed that PRKAR1B is significantly overexpressed in HNSC, with its elevated levels associated with poor prognosis and shorter survival. Growing evidence indicates that TME plays an essential role in facilitating tumorigenesis and progression. The results of this study conjointly suggest that PRKAR1B is correlated with immune cell infiltration within the TME. For instance, infiltration of M2 macrophages and activated mast cells was positively correlated with PRKAR1B expression. M2 macrophages exhibit tumor-promoting capabilities by secreting anti-inflammatory factors, encompassing TGF-β and IL-4, which facilitate immune suppression (20). They also secrete pro-angiogenic factors such as IL-8, CCL8, bFGF, and VEGF, thereby promoting angiogenesis and contributing to the biosynthesis of polyamines and proline (21). In turn, proline promotes extracellular matrix (ECM) remodeling, while polyamines are involved in cell proliferation. Furthermore, the role of M2 macrophages in therapy resistance is well documented across multiple cancer types (22, 23). Tumor cells frequently secrete stem cell factors that recruit mast cells, which subsequently release substances that contribute to angiogenesis, tissue remodeling, and immune regulation (24). Mast cells secrete pro-angiogenic molecules such as VEGF, bFGF, and heparin, and are key sources of ECM-remodeling proteases, aiding in tumor progression and metastasis (25).

It is worthwhile emphasizing that this study identified positive correlations between PRKAR1B expression and several genes, including C7orf50, EIF3B, TBRG4, DDX56, and BRAT1, which have been extensively researched in numerous disease contexts. C7orf50, a ubiquitin-related gene, plays a decisive role in nuclear ribosomal RNA assembly and is associated with the ubiquitination and degradation of estrogen receptor-α in breast and uterine cancers (26). EIF3B, a crucial component of the eukaryotic initiation factor family, plays a vital role in the assembly of the translation initiation complex. Besides, earlier studies have evinced that EIF3B promotes melanoma cell proliferation and migration by stabilizing PTGS2 expression and influencing liver cancer cell invasion and metastasis through the TGFBI/MAPK/ERK pathway (27). TBRG4 is a newly identified oncogene in breast cancer, with expression levels linked to malignancy, and also influences liver cancer cell proliferation and metastasis. DDX56, a member of the DDX RNA helicase family, is crucial for RNA metabolism and significantly contributes to the progression of various cancers, such as liver cancer and lung squamous cell carcinoma (28). BRAT1, involved in DNA damage response and mitochondrial homeostasis, has been implicated in neurodegenerative diseases and cancer, with its deficiency leading to misexpression of RNA and proteins, thereby affecting tumorigenesis (29). KEGG analysis exposed significant enrichment of pathways annotated as “Staphylococcus aureus infection” and “pancreatic secretion”. While these pathways may appear unrelated to HNSC, the enrichment of the Staphylococcus aureus infection pathway does not indicate actual infection in HNSC; rather, it reflects the aberrant activation status of immune cells, such as neutrophils, under PRKAR1B-associated regulation. Although these genes are categorized under infection-related response in KEGG, they fundamentally participate in remodeling the tumor immune microenvironment. Similarly, enrichment of the pancreatic secretion pathway suggests that the enriched genes may execute functions related to secretory regulation and matrix remodeling within the HNSC microenvironment.

Immune therapy has achieved significant progress in cancer treatment, with immune checkpoint blockade therapies extending survival in many cancers associated with poor prognosis. In clinical practice, inhibitors targeting PD-1/PD-L1 and CTLA-4 are extensively utilized. The results of the present study suggest that patients with high PRKAR1B expression may benefit from these therapies. Additionally, PRKAR1B expression was closely associated with the immune checkpoint genes CD276 and TNFRSF14, indicating that PRKAR1B could predict clinical responses to immune checkpoint blockade therapies. CD276, also termed B7-H3, is a member of the B7 family immune checkpoint molecules, is predominantly expressed in cancer cells and activated tumor-infiltrating immune cells (30). It aids immune evasion by suppressing the activity of cytotoxic T and NK cells and has emerged as a therapeutic target implicated in tumor growth, dissemination, and drug resistance (31). The gene encoding tumor necrosis factor receptor superfamily 14 (TNFRSF14), also referred to as HVEM, is linked to poor survival outcomes in various tumors due to its upregulation (32, 33). TNFRSF14 encodes a protein in the TNF receptor superfamily that triggers pro-inflammatory pathways. It mediates apoptosis and inhibits the immune escape mechanisms of tumor cells. Herein, the correlation between PRKAR1B expression and drug sensitivity was explored, and the results signaled that Lapatinib and Erlotinib were effective in the high-expression group. Lapatinib, an EGFR/HER2 inhibitor, has been widely studied for its efficacy in the treatment of breast, ovarian, and other cancers. In HNSC, Lapatinib, either alone or in combination with other drugs such as capecitabine, cisplatin, and paclitaxel, has shown promising results. Single-cell data revealed broad PRKAR1B expression within the CD8Tex subset, strongly implicating it in driving T-cell exhaustion. When integrated with the functional assays, these findings indicate that PRKAR1B accelerates tumor cell proliferation and suppresses apoptosis in a cell-autonomous manner while simultaneously fostering an exhausted CD8+ T-cell microenvironment, thereby impairing antitumor immunity. These complementary mechanisms act in concert to promote HNSC progression.

Moreover, the biological role of was validated in cellular experiments. Knockdown of PRKAR1B expression inhibited HNSC cell proliferation, as determined by CCK8 and EdU assay. Additionally, PRKAR1B silencing suppressed HNSC cell migration, as evidenced by the results of Wound healing and Transwell assay, potentially through modulation of EMT signaling pathways. Additionally, PRKAR1B protein was up-regulated in clinical HNSC samples. Taken together, these findings suggest that PRKAR1B contributes to HNSC development, consistent with the findings of bioinformatics analyses. Nevertheless, some limitations of this study merit acknowledgment. To begin, the conclusions were largely derived from public databases and self-collected samples; thus, multicenter cohorts are warranted to validate the prognostic role of PRKAR1B. Secondly, PRKAR1B expression was exclusively assessed in clinical samples, and its function was primarily examined in *in vitro* cell models and *in vivo* animal models. Deeper mechanistic investigations are necessitated to confirm the biological role of PRKAR1B in tumor growth and metastasis. Finally, conditional gene knockout models will be indispensable to clarify the role of PRKAR1B in tumor immunity.

## Conclusions

Through bioinformatics analysis and experimental validation, this study comprehensively explored the expression characteristics and prognostic value of PRKAR1B. The results demonstrated that PRKAR1B overexpression in HNSC was correlated with poor prognosis and altered tumor immune responses. Overall, this study highlights the clinical utility of PRKAR1B as a prognostic biomarker and therapeutic target, offering valuable insights for the development of future therapeutic strategies.

## Data Availability

The original contributions presented in the study are included in the article/**Supplementary Material**. Further inquiries can be directed to the corresponding author.
