# Supplementary material for: PRKAR1B as an oncogenic biomarker for diagnostic and prognostic stratification of tumor immunity, proliferation, and migration in head and neck squamous cell carcinoma
Source: Front Immunol. 2026 Feb 20;17:1770459. doi: 10.3389/fimmu.2026.1770459 (PMC12963008; doi:10.3389/fimmu.2026.1770459)
Supplement: Supplementary file 1 [file DataSheet1.docx]

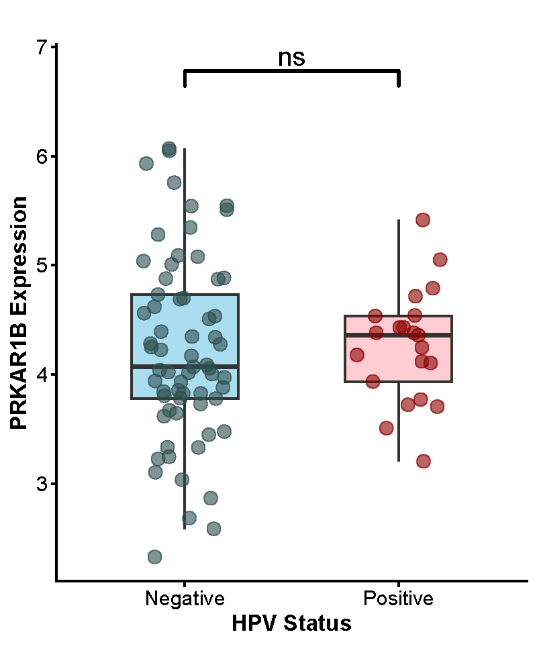


Figure S1 PRKAR1B expression in HPV-Negative and HPV-Positive samples from the TCGA database


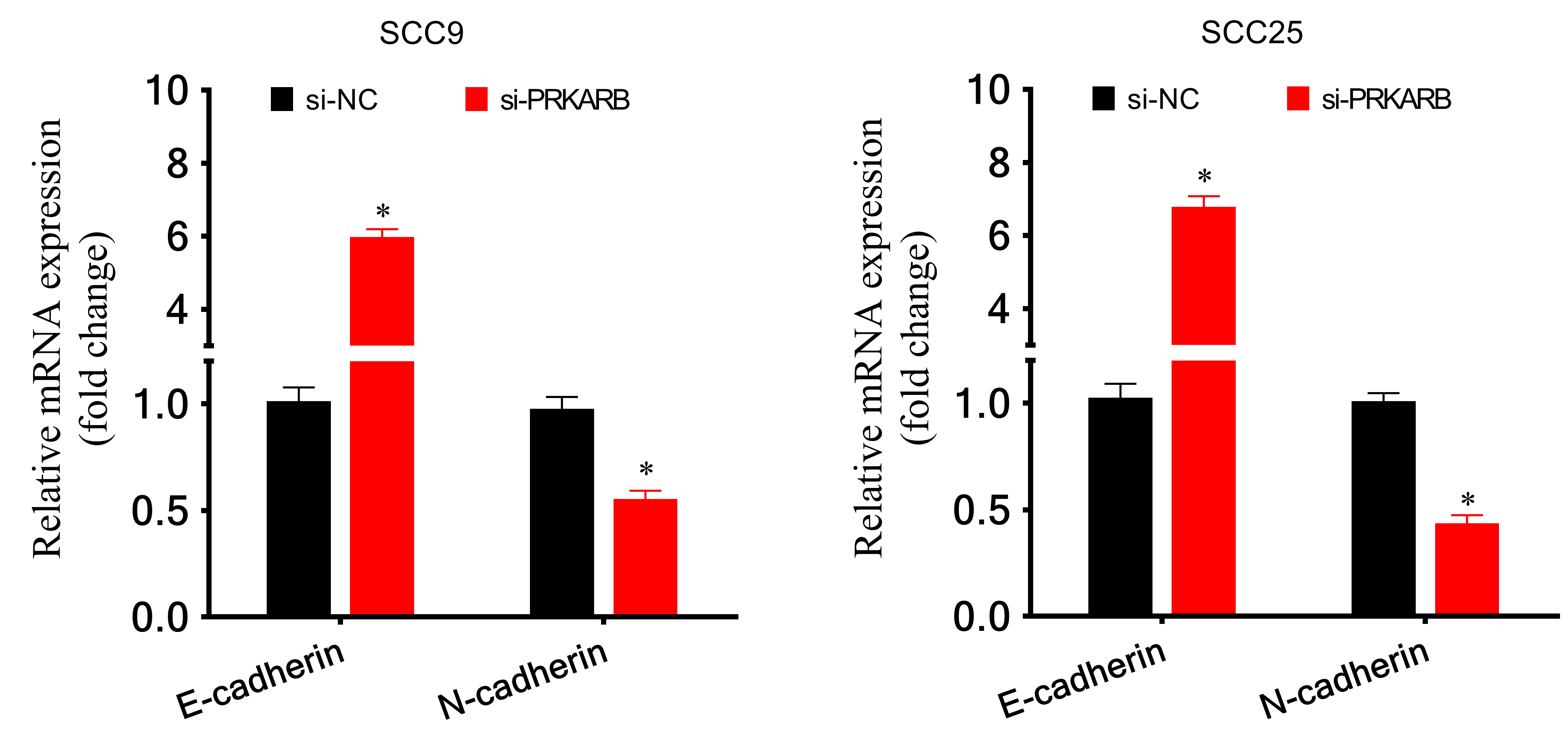


Figure S2 A and B. Relative expression levels of EMT markers in SCC9 and SCC25
